# Empirical validation of the diffusion model for recognition memory and a comparison of parameter-estimation methods

**DOI:** 10.1007/s00426-014-0608-y

**Published:** 2014-10-04

**Authors:** Nina R. Arnold, Arndt Bröder, Ute J. Bayen

**Affiliations:** 1 Institute for Experimental Psychology, Heinrich-Heine-Universität Düsseldorf, Universitätsstr. 1, 40225 Düsseldorf, Germany; 2 Present Address: School of Social Sciences, University of Mannheim, Schloss, Ehrenhof Ost, 68131 Mannheim, Germany

## Abstract

The diffusion model introduced by Ratcliff (Psychol Rev 85:59–108, 1978) has been applied to many binary decision tasks including recognition memory. It describes dynamic evidence accumulation unfolding over time and models choice accuracy as well as response-time distributions. Various parameters describe aspects of decision quality and response bias. In three recognition-memory experiments, the validity of the model was tested experimentally and analyzed with three different programs: fast-dm, EZ, and DMAT. Each of three central model parameters was targeted via specific experimental manipulations. All manipulations affected mainly the corresponding parameters, thus supporting the convergent validity of the measures. There were, however, smaller effects on other parameters, showing some limitations in discriminant validity.

## Introduction

Recognition tests are a widely used method to assess episodic memory performance. Previously presented (old) items must be distinguished from items that were not presented before (new items). It has been acknowledged early that in this paradigm, it is not trivial to derive good measures of memory from the correct responses (hits and correct rejections) and the erroneous ones (misses and false alarms, see e.g., Schulze, 1909). Model-based measures derived from signal detection theory (SDT; e.g. Snodgrass & Corwin, 1988) or from various threshold models disentangle memory performance from response biases (see Kellen, Klauer, & Bröder, 2013, for a discussion and comparison). These approaches, however, only model the result of cognitive processes ignoring how they unfolded over time. Ratcliff (1978) took a step further with his diffusion model describing the memory process as an accumulation of evidence until a threshold is reached. The model disentangles the memory measure further into two aspects that reflect objective processing (drift rate *v*) and a subjective achievement level (threshold parameter *a* and bias parameter *z/a*) (Wagenmakers, 2009). Accuracy data as well as reaction-time distributions of correct and false responses are used to estimate the model parameters, and speed–accuracy trade-offs are thus modelled.

The diffusion model (also Ratcliff diffusion model) was originally formulated for recognition memory and has been applied often in this domain (e.g., Ratcliff, 1978, 2006; Ratcliff, Thapar, & McKoon, 2004; Spaniol, Madden, & Voss, 2006; Thapar, Ratcliff, & McKoon, 2003). However, it has also been applied to many other binary-choice tasks, for example, in the areas of perception (Liu & Watanabe, 2012; Ratcliff, Thapar & McKoon, 2001, 2003, 2006b), prospective memory (Boywitt & Rummel, 2012; Horn, Bayen, & Smith, 2011, 2013; Rummel, Kuhlmann, & Touron, 2013), cognitive aging (McKoon & Ratcliff, 2012, 2013; Ratcliff, Thapar, & McKoon, 2001, 2003, 2004, 2006a, b, 2007; Spaniol et al., 2006; Spaniol, Voss, & Grady, 2008), post-error slowing (Dutilh, Forstmann, Vandekerckhove, & Wagenmakers, 2013; Dutilh et al., 2012), and in experiments involving response signals (Ratcliff & McKoon, 2008), a go/no-go task (Gomez, Ratcliff, & Perea, 2007), temporal-expectation effects on reaction time (Jepma, Wagenmakers, & Nieuwenhuis, 2012), task switching (Schmitz & Voss, 2012), priming (Voss, Rothermund, Gast, & Wentura, 2013), and the Implicit Association Test (Klauer, Voss, Schmitz, & Teige-Mocigemba, 2007). It has also been applied to clinical problems such as aphasia and dyslexia (Ratcliff, Perea, Colangelo, & Buchanan, 2004), depression (Pe, Vandekerckhove, & Kuppens, 2013), and to the impact of sleep deprivation on cognitive performance (Ratcliff & Van Dongen, 2009). Thus, the diffusion model has a wide area of applications. For an overview, see Ratcliff and McKoon (2008).

Although there is some existing evidence supporting the diffusion model’s validity as discussed below, a systematic experimental validation of model parameters in the recognition-memory domain has not been performed to date. We conducted validity tests in three recognition experiments each targeting one of the core model parameters. Additionally, we analyzed our experimental data with three computer programs to compare different methods for parameter estimation: fast-dm (Voss & Voss, 2007), EZ (Wagenmakers, Van der Maas, & Grasman, 2007), and DMAT (Vandekerckhove & Tuerlinckx, 2008). We will first describe the diffusion model and its parameters in detail as well as approaches for parameter estimation.

## The diffusion model

The diffusion model is designed for fast binary choices (with mean reaction times faster than about 1,500 ms). It utilizes the information available from the participant’s responses in the best possible way. That is, it considers not only mean reaction times and accuracy, but also relative speed of false and correct responses, and the shape of reaction-time distributions (Ratcliff et al., 2004).

**Fig. 1:**
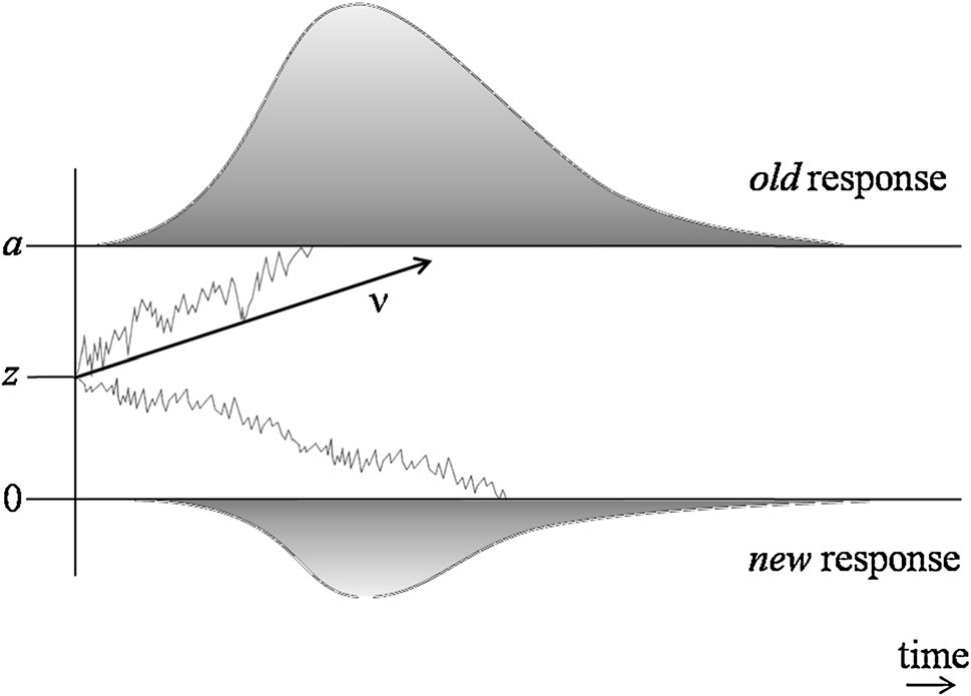
Schematic illustration of the diffusion model. The process starts at the starting point *z* and accumulates information over time until one of two thresholds is reached. The speed of information accumulation is indicated by the drift rate *v*. Due to random influences the process is not linear, but fluctuates between the thresholds. The upper threshold *a* is associated with the old response, the lower threshold 0 is associated with the new response. As soon as a threshold is reached the corresponding response is initiated. Adapted from “An illustration of the random walk and diffusion process, together with relatedness distributions that drive the diffusion process” by Ratcliff (1978), A theory of memory retrieval. *Psychological Review*, 85, p. 64, and “Schematic illustration of the diffusion model” by Voss et al. (2004), Interpreting the parameters of the diffusion model: an empirical validation. *Memory & Cognition*, 32, p. 1207

The main idea underlying the diffusion model is shown in Fig. 1. Confronted with a binary choice task like old–new recognition, a participant will start accumulating internal evidence for the decision. Depending on the relative amounts or quality of information favoring one of the options, the evidence will drift to one of two decision boundaries, and the process will terminate in a decision for one option when one of the boundaries is crossed. The drift towards a boundary is modelled as a diffusion process, which is the continuous generalization of a random walk. There are slight differences in parameter labels in the literature. We use the labels used by Voss and Voss (2007, 2008). However, they are easily translated to other labels.

The drift rate *v* represents the quality of the information extracted from the stimuli (Ratcliff et al., 2004). The drift rate *v* depends on the degree of match between a memory probe and information stored in memory (Ratcliff, 1978; Ratcliff & Starns, 2009). It describes the information accumulation per time unit and is, therefore, the average gradient, that is, the mean rate of approach to one of the thresholds. Positive values indicate an approach to the upper threshold, whereas negative values indicate an approach to the lower threshold. The absolute value describes the speed of information accumulation. The higher the absolute value is, the faster the corresponding threshold is reached, and the less likely it is that the response opposite to the drift rate––which is often wrong––is chosen (Voss, Rothermund, & Voss, 2004). Every item in a recognition test has its own drift rate. The drift rate is assumed to be normally distributed with mean *v* and standard deviation *s*_*v*_ (Ratcliff et al., 2004).

The distance between the decision boundaries (threshold parameter *a*), on the other hand, defines how much information a participant needs before making a decision (Voss et al., 2004). The upper threshold *a* is the criterion for responding old in a recognition-memory test. Conventionally, the lower boundary is set to zero. Therefore, the value of the upper threshold *a* is a measure of the distance between the thresholds. Obviously, the decision boundaries also affect accuracy, because wider distances reduce the probability of the process diffusing across the “wrong” boundary by chance. Thus, *v* and *a* both contribute to performance, and *a* is believed to be affected by speed–accuracy trade-offs (Ratcliff, 1978). A higher threshold parameter *a* indicates that a person needs more information to make a decision. This leads to a higher rate of correct, but on average slower, responses (Ratcliff et al., 2004).

The starting point *z* of information accumulation describes possible asymmetries in the amount of information that is needed to exceed the response criteria for old versus new responses. If *z* equals *a*/2, there is no bias towards one response or the other. If *z* differs from *a*/2, the reaction times for old versus new responses will differ. The smaller the distance between starting point and threshold, the lower the reaction times will be. If *z* > *a*/2, less information is needed to exceed the upper threshold *a*. Thus, there is a bias towards the old response. If *z* < *a*/2, there is a bias towards the new response. It is assumed that the starting point varies between trials with a uniform distribution with mean *z* and range *s*_*z*_ (Ratcliff, 1978).

The model does not assume a linear process, but takes into account random influences that add to the constant influence of the drift rate. This explains why processes with the same drift rate can have different reaction times or even opposite responses (Ratcliff & Rouder, 1998). Random influences at time *t* are described by a normal distribution with mean 0. The variance increases with time. Increase in variance is represented by the diffusion constant *s*. *s* is a scaling parameter, fixed to any positive value.

In addition, there are other processes contributing to reaction time, such as motor processes and stimulus encoding. In the model, their total time is estimated as the response-time constant *t*_0_. It contains the non-decisional proportion of the reaction time (Ratcliff, 1978). The total reaction time RT equals RT_decision_ + *t*_0_. Like drift rate *v* and starting point *z*, this parameter differs between trials. *t*_0_ is uniformly distributed with range *s*_*t*_ (Ratcliff et al. 2004). Ratcliff (2013) showed that, in most cases, these standard assumptions about the distributions of drift rate, starting point, and response-time constant lead to the same predictions as different distributional assumptions.

Since 1978, when the diffusion model was introduced, there have been some modifications in the use of the model. Ratcliff (1978) postulated that the process is self-terminating for matches, but exhaustive for non-matches. This implies that there are two different processes for the two boundaries. The upper boundary is reached when a match is found, and all other processes are then terminated. For the lower boundary, all processes must result in a non-match. According to Ratcliff (1978), recognition is best described by parallel processes. For each item in the search set, a comparison with the memory probe is running. The observed reaction time reflects only the maximum (for non-matches) or the minimum (for matches) of the diffusion processes (Ratcliff, 1978, 1988).

The model has also been used in paradigms other than recognition memory, where the assumption of a difference between the two boundaries is unnecessary because only one simultaneous comparison is assumed. In later descriptions of the diffusion model, there is no differentiation between descriptions of the diffusion model for recognition-memory experiments and descriptions of the diffusion model for other tasks. According to these descriptions, the response is initiated as soon as a boundary is reached (Ratcliff et al., 2004, 2007; Spaniol et al. 2006, 2008; White et al. 2009). Recently, the drift criterion has attracted some attention. The drift criterion can be seen as the zero point of the drift rate. It describes the amount of evidence above which evidence accumulates towards the upper threshold and below which evidence accumulates toward the lower threshold (Criss, 2010; Ratcliff, 1978, 1981, 1985, 1987; Ratcliff, Van Zandt, & McKoon, 1999). However, this parameter is not implemented in the available programs.

## Data analysis and parameter estimation with the diffusion model

The aim of the parameter estimation is to find the optimal fit between theoretical and empirical reaction-time distributions and accuracy data. Therefore, formulas for the probability density functions (PDF) or the cumulative distribution function (CDF) for both thresholds are needed. For a detailed description and discussion of this topic, see Tuerlinckx, Maris, Ratcliff, and De Boeck (2001), Tuerlinckx (2004), and Ratcliff and Tuerlinckx (2002). To estimate the parameters, a criterion for the goodness-of-fit is needed. For a discussion of different criteria see Read and Cressie (1988), Ratcliff and Tuerlinckx (2002), and Voss et al. (2004). The parameter estimation of the diffusion model has no analytical solution. Therefore, to find the best fit, numerical integration procedures are implemented (Wagenmakers et al., 2007). Parameter estimation is quite complex and is a research topic of its own (Diederich & Busemeyer, 2003; Ratcliff & Tuerlinckx, 2002; Tuerlinckx, 2004; Wagenmakers et al., 2007; Vandekerckhove & Tuerlinckx, 2007). In recent years, some programs have been developed to make the diffusion model easy to use: EZ-diffusion model (Wagenmakers et al., 2007), DMAT (Vandekerckhove & Tuerlinckx, 2008), and fast-dm (Voss & Voss, 2007). Vandekerckhove et al. (2011) developed a hierarchical extension of the diffusion model.

Van Ravenzwaaij and Oberauer (2009) tested the parameter recovery of fast-dm, DMAT, and EZ with simulated data. They calculated correlations between the true values and the estimated parameter values. All methods were able to estimate the parameters with reasonable accuracy. Fast-dm seemed to be the least robust method for parameter estimation. This was due to an incapability of recovering individual differences for the dispersion parameters s_v_ and s_z_, and a tendency to yield smaller differences between conditions, especially for the drift rate, with a small number of trials.

DMAT requires a large number of trials. In contrast, EZ and fast-dm provide useful estimates with about 80 trials per condition (Van Ravenzwaaij & Oberauer, 2009). In our experiments, we had a relatively small number of trials per condition because we wanted to mimic standard conditions of a recognition-memory experiment. For a small number of trials, Van Ravenzwaaij and Oberauer (2009) found that EZ was most robust. However, the parameter *z* is fixed in this model, and since we also wanted to validate this bias parameter, we used fast-dm and DMAT to estimate the parameters and cross-checked the results with EZ for the two experiments not targeting the bias parameter (Experiments 2 and 3).

The EZ-diffusion model (Wagenmakers et al., 2007) is an algorithm that was developed to make data analyses with the diffusion model as easy as possible. It transforms accuracy and the mean and variance of the reaction times of correct responses into drift rate *v*, threshold parameter *a*, and response-time constant *t*_0_ via three equations. As an advantage, these equations do not require any parameter fitting and can be used even if the error rate is very small. To achieve this, the model makes some simplifications. That is, (1) it assumes there is no between-trial variability, and thus, *s*_*v*_, *s*_*z*_ and *s*_*t*_ are set to zero. (2) The starting point is assumed to be unbiased, and thus, *z*/a is set to 0.5.

Fast-dm (Voss & Voss, 2007) uses the partial differential equation (PDE) method to compute the CDF (Voss & Voss, 2008) and the Kolmogorov–Smirnov test (KS test; Kolmogorov, 1941) to estimate the parameters and determine the model fit. The PDE method avoids infinite sums and has the advantage of evaluating all starting points at the same time, thus reducing computing time (Voss & Voss, 2008). The KS test uses the test statistic *T* as the optimization criterion, and parameters are chosen such that *T* is minimized. The reaction-time distributions of both thresholds are estimated together by giving the reaction times of the lower threshold a negative sign. The parameter space is searched via the simplex method (Nelder & Mead, 1965) to obtain the best model fit. Starting points for *v*, *a*, and *t*_0_ are provided by the EZ model (Wagenmakers et al., 2007). Realistic values are chosen as starting points for the other parameters.

The diffusion model analysis toolbox (DMAT; Vandekerckhove & Tuerlinckx, 2008) is a Matlab toolbox with a graphical user interface. It uses design matrices to obtain parameter estimates. Chi-square and maximum-likelihood estimates are available for parameter estimation and goodness-of-fit tests.

As described above, fast-dm and DMAT use different test statistics. Each statistic has several advantages and disadvantages, and the authors of the programs motivated the choice of their statistics differently. Voss and Voss (2007) chose the KS test because it does not aggregate data and, thus, does not lose information. Additionally, it is not affected by outliers as much as the maximum-likelihood and the Chi-square statistic. The Chi-square statistic is more robust and faster than the maximum-likelihood method (Ratcliff & Tuerlinckx, 2002). Chi-square and maximum-likelihood methods are commonly used for parameter estimation.

In applications of the diffusion model reported by Ratcliff and colleagues, *s* was usually set to 0.1 (e.g., Ratcliff, 1978, 1988, 2002; Ratcliff & Rouder, 1998, 2000; Ratcliff et al., 2001, 2003, 2004, 2006a, b, 2007). DMAT and EZ set *s* = 0.1 by default. In applications of the model reported by Voss and colleagues, *s* was usually set to 1 (Voss et al., 2004; Spaniol et al. 2006, 2008). The fast-dm program (Voss & Voss, 2007) also uses a diffusion constant of 1. However, parameters that were obtained via computations based on other diffusion constants can simply be transformed by multiplying all parameters (except *t*_0_) by the desired diffusion constant. We converted the fast-dm results to *s* = 0.1 to make the results more comparable.

## The validity of the model

When first publishing the diffusion model, Ratcliff applied it to several recognition-memory paradigms including the old–new paradigm used here. He showed that the drift rate accounted for primacy and recency effects (Ratcliff, 1978). Since 1978, the model has been applied in many studies of recognition memory, yielding insights into the underlying dynamics of the process (e.g., Ratcliff, 1978, 2006; Ratcliff et al., 2004; Spaniol et al., 2006; Thapar et al., 2003) and having far-reaching implications, for example falsifying the global slowing hypothesis of cognitive aging (Wagenmakers, 2009).

There are also neuroscientific studies that support the model’s fit to data. Ratcliff, Cherian, and Segraves (2003) examined macaques via the moving-dot paradigm. In this paradigm, there are several dots moving randomly. Among them, however, are some dots that move simultaneously. The task is to identify the dots that move simultaneously. Ratcliff et al. (2003) showed that the macaques’ behavior as well as their neuronal activity could be fitted by the diffusion model. The fit of behavioral data from the moving-dot paradigm (Julesz, 1971) was also shown for humans (Ratcliff & McKoon, 2008).

These studies supported the model because the model fitted the data well, and they were able to explain a range of phenomena. However, interpreting parameter estimates as measures of cognitive processes requires construct validity of the measurement model in the sense of Cronbach and Meehl (1955). That is, the measures must show convergent as well as discriminant validity. Convergent validity is assessed by a measure’s covariation with related constructs, whereas discriminant validity refers to the lack of covariation with unrelated constructs. Measures are “process-pure” to the extent they show both types of validity. A systematic experimental validation assessing both types of validity is lacking in the realm of recognition memory. Parameter estimates are mathematical abstractions, and a systematic empirical justification of their psychological interpretation is indispensable.

In the perceptual domain, a systematic experimental validation of the diffusion model was conducted by Voss et al. (2004), using a color discrimination task. In a first experiment, Voss et al. manipulated variables to affect the drift rate *v*, the threshold parameter *a*, and the response-time constant *t*_0_. Their participants had to decide whether a dot stimulus was dominated by orange or by blue dots. There were four conditions, namely one standard condition and three other conditions that each targeted one specific model parameter. Task difficulty was increased to decrease the drift rate (difficult condition). An instruction to be very accurate was aimed at increasing the threshold parameter *a* exclusively (accuracy condition). Finally, by allowing participants to press the response keys with one finger only, the authors strove to increase the response-time constant *t*_0_ (handicap condition). They found the predicted pattern. That is, higher task difficulty decreased drift rate, accuracy instructions led to a higher threshold parameter, and the handicap condition led to an increased response-time constant *t*_0_. However, the authors also found unexpected results. In the accuracy condition, the *t*_0_ parameter was higher than in the standard condition. In the handicap condition, the drift rate for blue dominated stimuli *v*_blue_ and the starting point *z*/*a* differed significantly from those in the standard condition. The increased *t*_0_ parameter was easily explained because if participants have more time to respond they execute their responses more slowly. Differences in drift rate and starting point in the handicap condition, however, could not be explained that easily. However, all individual models revealed good model fit as assessed via the goodness-of-fit statistic *T* (see Voss et al., 2004, for a detailed description). In a second experiment, Voss et al. (2004) manipulated the starting point by promoting one response over the other. They found that the starting point was biased towards the promoted response. Overall, the models described the empirical data well. The authors concluded that the parameters of the diffusion model represent the process components of the perceptual task well. The study supported the convergent and partly the discriminant validity of the diffusion-model parameters in the perceptual domain.

Additional support for the model’s validity came from Ratcliff and Rouder (1998) for psychophysical tasks and from Wagenmakers, Ratcliff, Gomez, and McKoon (2008) for the lexical-decision task. They showed that accuracy instructions increased the threshold parameter *a*, and that easier stimuli have higher drift rates. Wagenmakers et al. (2008) showed that unequal presentation proportions affected not only the starting point but also the boundary separation.

The aim of the present study was to provide a similar test of the model’s validity in the recognition domain. In this article, we present three recognition-memory experiments each targeting one central model parameter. In Experiment 1, we manipulated the ratio of old to new items in the test (targeting bias parameter *z*). In Experiment 2, we manipulated the instructions for accuracy versus speed (targeting threshold parameter *a*). In Experiment 3, we used a manipulation to affect the quality of encoding (targeting drift-rate parameter *v*).

If each manipulation affects the predicted parameter in the expected direction without influencing other parameters, this would be strong support for the validity of the model. Therefore, we tested if experimental manipulations targeting the process components of the diffusion model affected the corresponding parameters (convergent validity) and only these (discriminant validity).

Ratcliff (1978) advised against between-subject designs because in such designs, differences in reaction times may be due to between-group differences in speed–accuracy criteria (threshold parameter *a*). However, some variables cannot be experimentally manipulated within participants, such as, for example, the age variable in studies of cognitive aging (e.g., Spaniol et al., 2006). Hence, it is useful to know if the model is valid for both types of design. We, therefore, tested model validity using within-subject designs (Experiment 3) as well as between-subject designs (Experiments 1 and 2).

We analyzed the data with three different methods: fast-dm (Voss & Voss 2007), EZ (Wagenmakers et al., 2007), and DMAT (Vandekerckhove & Tuerlinckx, 2008). Van Ravenzwaaij and Oberauer (2009) compared these methods with simulated data. For individual differences, they found that EZ did better than fast-dm and DMAT, and that there was no consistent difference between fast-dm and DMAT regarding the correlation with the true values that generated the data. Fast-dm and DMAT both had difficulties with the dispersion parameters which are not estimated by EZ. Regarding parameter means, EZ showed a small bias to underestimate drift rate and non-decision time and to overestimate the threshold parameter. However, it covered the mean structure of the data and showed mean parameter differences between conditions in the expected direction. DMAT showed the smallest bias, but underestimated response-time constant *t*_0_, and overestimated drift rate and boundary separation. It covered group differences well. Fast-dm showed the largest bias and showed smaller group differences than there were in the simulated data sets. Van Ravenzwaaij and Oberauer concluded that all three methods show reasonable accuracy when they have sufficient data points. DMAT required a large number of data points, whereas EZ and fast-dm needed only 80 data points to produce reasonable estimates. EZ and DMAT proved better at detecting group differences. Thus, it is not easy to decide which toolbox to use. EZ seems to be very accurate but cannot detect differences in the bias parameter. DMAT is better than fast-dm at detecting group differences but needs more trials to yield reasonable estimates.

The aim of our study is similar to that by Voss et al. (2004) in that we experimentally evaluated the validity of the diffusion model. While Voss and colleagues validated the model in the perceptual domain, we evaluated its validity for recognition-memory experiments. There is no a priori reason to believe that perceptual evidence accumulation and retrieval from memory follow the same laws. Hence, an assessment of construct validity is necessary in both domains. Additionally, our work is similar to the work by van Ravenzwaaij and Oberauer (2009) in the sense that it compares different methods for estimating diffusion-model parameters. Unlike van Ravenzwaaij and Oberauer, we did not simulate data but we analyzed our data with all three toolboxes to perform a systematic comparison of the three methods with experimental data. Our experiments were typical recognition experiments and did, hence, not provide perfect conditions for data analysis with the diffusion model. For example, we used relatively few trials (resulting in relatively few error responses) compared with a lexical-decision task or a perceptual task. Hence, we examined the performance of the three methods in compromised fitting situations.

## Experiment 1

The first experiment tested the validity of the starting-point parameter *z*, using a standard response-bias manipulation, namely the manipulation of the ratio of old to new items in the test (e.g., Bröder & Schütz, 2009; Criss, 2010; Macmillan & Creelman, 2005; Starns, Ratcliff, & McKoon, 2012). Participants were informed about this ratio. Words were used as stimuli. We expected the ratio manipulation to affect the bias parameter *z*/*a*, exclusively. If there are more old words than new words in the test––and participants are aware of this––the starting point is expected to be biased towards the threshold for the old response. Accordingly, if there are more new words than old words in the test, the starting point is expected to be biased towards the threshold for the new response. If this manipulation specifically affects the bias parameter and not the other parameters, this would provide strong support for the diffusion model.

This response-bias manipulation was used by Rotello et al. (2006), for example. They found that participants adopted a lenient signal detection criterion when they were informed that the majority of the test items were old. The signal detection criterion resembles the bias parameter *z*/*a* of the diffusion model (Ratcliff & McKoon, 2008). Hit rate and false-alarm rate both increase as the proportion of old items increases (Criss, 2010; Rotello et al., 2006). Bröder and Schütz (2009) showed that this manipulation affected the bias parameters in SDT and a two high-threshold model in a similar fashion.

### Methods

#### Participants

60 participants (53 female) took part in the experiment. They were students at the University of Düsseldorf (*M* (age) = 22.3 years, range 18–35 years) who received course credit or monetary payment.

#### Design

We manipulated the ratio of old to new items between participants with two levels (1:2 versus 2:1).

#### Materials

Items were drawn from a pool of 285 nouns that we selected from a collection of German nouns normed for concreteness (Hager & Hasselhorn, 1994). The ratings vary between −20 (very abstract) and +20 (very concrete). Our pool included 285 concrete nouns (mean ratings >+5) of 4–9 letters.

#### Procedure

There were one or two participants in each session, seated in individual computer booths. Stimulus presentation and response recordings were computer directed. For each participant, 140 nouns were randomly drawn from the pool for the study list. They were presented one at a time for 2 s each in the center of the screen, preceded by a primacy buffer of five items that were the same for all participants. Participants were instructed to concentrate on the words and to memorize them. After a three-minute filler task (mental rotation), the test phase followed. In the old-bias condition, there were 140 old nouns (i.e., all nouns from the study list) and 70 new nouns (randomly drawn from the remaining items in the pool). In the new-bias condition, there were 70 old nouns (randomly drawn from the study list) and 140 new nouns. Participants were informed about the number of old and new words before the test phase started. To ensure understanding of the instructions, participants were asked if there were more old words or more new words in the test. All participants could answer this question correctly. Two marked keys on the keyboard (*C* and *M*) were used for the responses in the test. The assignment of the keys to the response options old and new was counterbalanced across participants. Three seconds after response selection, the next item appeared on the screen. If the latency of a response exceeded 4 s, a reminder appeared on the screen prompting the participant to respond faster. After completion of the recognition test, participants were debriefed. The average length of a session was approximately 45 min.

### Results

#### Performance measures

Mean hit rates were 0.61 (SD = 0.14) in the *new*-*bias* condition and 0.71 (SD = 0.14) in the *old*-*bias*-condition, a significant difference, *t*(58) = −2.45, *p* = 0.02, *d* = 0.63. False-alarm rates were 0.17 (SD = 0.10) in the new-bias condition and 0.29 (SD = 0.15) in the old-bias condition, also a significant difference, *t*(58) = −3.65, *p* < 0.01, *d* = 0.94. The two groups did not differ in terms of SDT’s sensitivity parameter *d*′ (*M* (new-bias) = 1.35, SD (new-bias) = 0.54; *M* (old-bias) = 1.22, SD (old-bias) = 0.64) but differed significantly in the response criterion *c* (*M* (new-bias) = 0.38, SD (new-bias) = 0.41; *M* (old-bias) = 0.02, SD (old-bias) = 0.35), *t*(58) = 3.65, *p* < 0.01, *d* = 0.94. Mean reaction times showed no significant differences. They were 945 ms (SD = 0.14) in the new-bias condition and 978 ms (SD = 0.14) in the old-bias condition.

#### Parameter estimation and model fit

First, we performed parameter estimation and goodness-of-fit tests with the fast-dm program (Voss & Voss, 2007) and with DMAT (Vandekerckhove & Tuerlinckx, 2008). For each participant, we calculated one model with two different drift rates––one for old and one for new items. Each model was based on 210 trials (for the drift rates there were 140 and 70 trials, respectively). Following Voss et al. (2004), we excluded trials with reaction times below 300 ms and above 4,000 ms from analyses because Ratcliff and Tuerlinckx (2002) showed that outliers may have a strong effect on parameter estimation, and because after 4,000 ms, participants were reminded to answer faster. We excluded a total of 37 trials (<1 %). The upper threshold was associated with the old response; the lower threshold was associated with the new response and was set to 0. Thus, negative drift rates indicate an approach toward the new response, whereas positive drift rates indicate an approach toward the old response.

We estimated eight parameters per participant: the mean bias parameter *z*, the mean upper threshold *a*, the mean drift rate for old items *v*_old_, the mean drift rate for new items *v*_new_, the mean response-time constant *t*_0_, the range of the bias parameter *s*_*z*_, the range of the response-time constant *s*_*t,*_ and the standard deviation of the drift rates *s*_*v*_. Like Voss et al. (2004), we present *z*/*a* instead of *z* because *z*/*a* is easier to interpret. A bias parameter of *z*/*a* = 0.5 represents an unbiased starting point. Values greater than 0.5 indicate a bias towards the old response; values lower that 0.5 indicate a bias towards the new response.

For fast-dm, the KS test showed a good fit for all individual models (*p* > 0.05). For DMAT, we used the Chi-square method with default bins to estimate parameters and to calculate the model fit. The Chi-square test showed good model fit for 57 models and bad model fit for the remaining three individual models. We only included models with sufficient model fit (i.e., *p* > 0.05). Since some participants made very few mistakes, we encountered several warnings with DMAT. However, we included the parameter estimates in the analysis when they had reasonable fit. As this experiment was designed to target the bias-parameter *z*/*a*, we did not analyze the data with the EZ method because in EZ, *z*/*a* is set to 0.5.

#### Parameter analyses with fast-dm

The significance level was set to 0.05 for all our tests. Drift rates were significantly steeper for new items than for old items in both conditions (new-bias: *M* (old) = 0.05, SD (old) = 0.08, *M* (new) = 0.14, SD (new) = 0.06, *t*(29) = −5.60, *p* < 0.01, *d* = 1.02; old-bias: *M* (old) = 0.04, SD (old) = 0. 05, *M* (new) = 0.14, SD (new) = 0.07, *t*(29) = −6.41, *p* < 0.01, *d* = 1.17). To test the influence of the manipulation, we conducted independent-samples *t* tests for each parameter. As predicted, the bias-parameter *z*/*a* was significantly higher in the old-bias condition than in the new-bias condition, *M* (old-bias) = 0.66, SD (old-bias) = 0.09, *M* (new-bias) = 0.49, SD (new-bias) = 0.10, *t*(58) = −7.11, *p* < 0.01, *d* = 1.84. As *z*/*a* = 0.5 represents an unbiased starting point, both conditions should differ significantly from this neutral point. The bias parameter for new items was not significantly different from 0.5, *t*(29) = −0.69, *p* = 0.49, *d* = 0.01, which suggests that contrary to prediction there was no bias in the starting point. In the old condition, the bias-parameter was significantly higher than 0.5, *t*(29) = −9.86, *p* < 0.01, *d* = 1.78, as predicted.

**Fig. 2:**
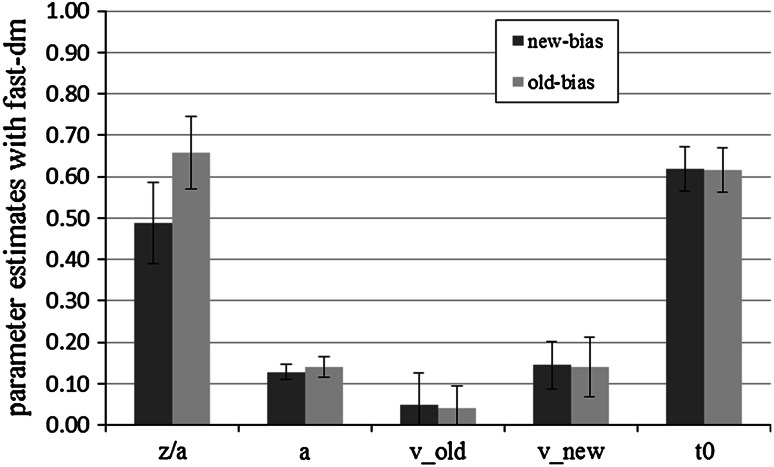
Mean fast-dm parameter estimates for new-bias and old-bias conditions in Experiment 1. *Bars* represent standard deviation. We show the absolute values of the drift rates. *z*/*a* represents the bias parameter, *a* the threshold parameter, *v*_old_ the drift rate for old items, *v*_new_ the drift rate for new items, and *t*_0_ the response-time constant

Contrary to predictions, the threshold parameter *a* also differed significantly between conditions, *M* (old-bias) = 0.14, SD (old-bias) = 0.02, *M* (new-bias) = 0.13, SD (new-bias) = 0.02, *t*(58) = −2.13, *p* = 0.04, *d* = 0.55. Participants in the old-bias condition showed a larger value of the threshold parameter than participants in the new-bias condition. Thus, the former were more conservative. The effect size (measured by Cohen’s *d*), however, was only about one-third of that of the bias parameter. No other comparison yielded significance (all *p* > 0.05). Averaged mean parameter estimates are shown in Fig. 2.

#### Parameter analyses with DMAT

**Fig. 3:**
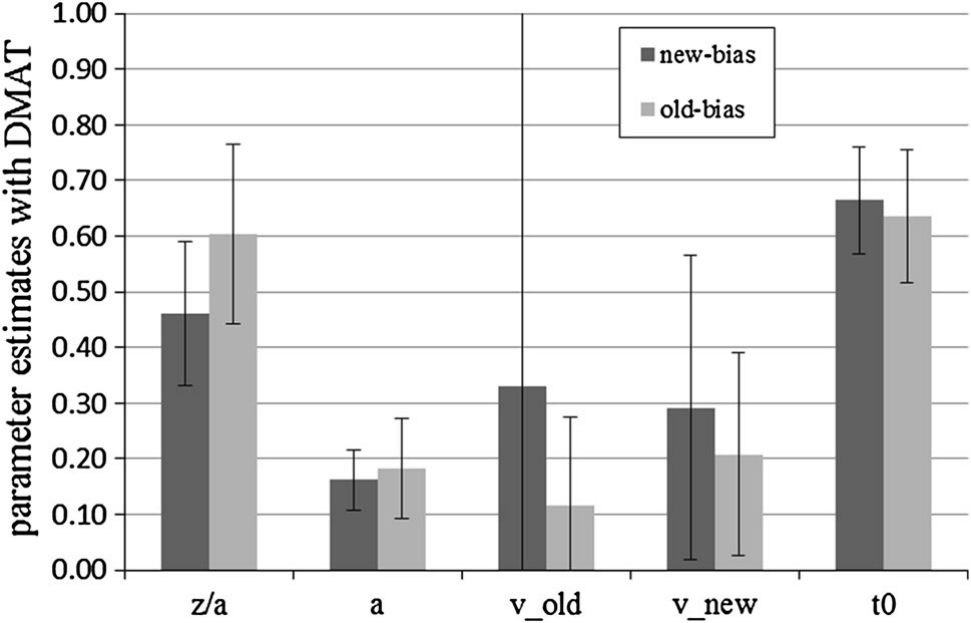
Mean DMAT parameter estimates for new-bias and old-bias conditions in Experiment 1. *Bars* represent standard deviation. We show the absolute values of the drift rates. *z*/*a* represents the bias parameter, *a* the threshold parameter, *v*_old_ the drift rate for old items, *v*_new_ the drift rate for new items, and *t*_0_ the response-time constant

There was no significant difference between the absolute value of the drift rates for old and new items in either condition (all *p* > 0.05) Again, the bias-parameter *z*/*a* was significantly higher in the old-bias condition than in the new-bias condition, *M* (old-bias) = 0.60, SD (old-bias) = 0.16, *M* (new-bias) = 0.46, SD (new-bias) = 0.13, *t*(58) = −3.70, *p* < 0.01, *d* = 0.99. The bias parameter of the old-bias condition differed significantly from 0.5, *p* < 0.01, *d* = 0.65, but the bias parameter in the *new*-*bias* condition did not, *p* = 0.12, *d* = 0.31. No other comparison yielded significance (all *p* > 0.05). Averaged mean parameter estimates are shown in Fig. 3.

### Discussion

We conducted this experiment to validate the interpretation of the bias parameter *z*/*a* of the diffusion model. We manipulated the proportion of old to new items at test. This should affect the bias parameter and have no effect on other parameters. In line with the hypothesis, the manipulation affected the bias parameter most strongly according to both estimation methods. The effect size *d* was large to very large in each case according to Cohen’s (1988) conventions. However, the manipulation also had a medium-sized effect on the threshold parameter as estimated with fast-dm.

The bias parameter *z*/*a* is the starting point of the diffusion process. Along with the thresholds (parameter *a*) it defines the amount of information that is necessary to make a decision to call the item old or new. When there were more old items in the test, the starting point moved towards the upper threshold, but at the same time the thresholds moved apart. Whether the effect on parameter *a* is a genuine psychological effect of stricter criteria or rather a problem of missing discriminant validity of the model parameters estimated with fast-dm cannot be decided at this point. If it were the former, it would underline Ratcliff’s (1978) warning against between-subjects designs which may lead to differing criteria in the experimental conditions, although in this case for unknown reasons.

To summarize, in Experiment 1, both estimation methods showed convergent validity and found the predicted difference in the starting point. However, only DMAT showed satisfying discriminant validity. Fast-dm found unpredicted differences in one other parameter, although this effect was considerably smaller.

## Experiment 2

In Experiment 2, we used the same materials and similar procedures as in Experiment 1. The aim of this experiment was to test the validity of the threshold parameter *a*. Participants received different kinds of feedback depending on their experimental condition. In the accuracy condition, participants received negative feedback if they made a mistake. In the speed condition, participants received negative feedback if they responded more slowly than within 1,000 ms. This manipulation was expected to lead to an adjustment of thresholds. Participants in the accuracy condition should adopt more conservative criteria and thus have a higher threshold parameter than participants in the speed condition. Ratcliff et al. (2004) used a similar manipulation as a within-subject manipulation, but they fixed the other model parameters between the conditions and compared the results for young and older adults. They showed that the model captured the effect of speed and accuracy instructions with only threshold parameter *a* changing.

### Methods

#### Participants

There were 60 participants (49 females) between 18 and 35 years (*M* = 22 years) in the experiment, 59 students from the University of Düsseldorf and one employee. They participated for course credit or monetary payment.

#### Design

The speed/accuracy instructions were manipulated between participants.

#### Materials

We used the same nouns as in Experiment 1.

#### Procedure

There were one or two participants in each session. They were randomly assigned to the conditions. Study phase and distractor task were the same as in Experiment 1. The test items consisted of the 140 randomly chosen nouns presented during the study phase and 140 new nouns. Assignments of marked keys to responses were the same as in Experiment 1. The conditions differed only in the test phase. Depending on condition, participants received negative feedback, either on responses that were inaccurate or on responses that were too slow. Participants in the speed condition received negative feedback when they responded too slowly, that is not within 1,000 ms. The speed feedback screen reminded participants to respond within 1,000 ms and showed how long their response-time had been. Participants in the accuracy condition received negative feedback if their response was wrong. They were reminded to respond as accurately as possible. In each condition, the negative feedback was given in black font on a glaring red background. It stayed on the screen for 4,000 ms. No other feedback was provided.

### Results

#### Performance measures

Mean hit rates were 0.61 (SD = 0.15) in the speed condition and 0.69 (SD = 0.14) in the accuracy condition, a significant difference, *t*(58) = −2.08, *p* = −0.04, *d* = 0.54. False-alarm rates were 0.29 (SD = 0.15) in the speed condition and 0.29 (SD = 0.13) in the accuracy condition (n.s.). The two groups neither differed in terms of SDT’s sensitivity parameter *d*′ [*M* (speed) = 0.93, SD (speed) = 0.62; *M* (accuracy) = 1.14, SD (accuracy) = 0.73] nor in the response criterion *c* [*M* (speed) = 0.16, SD (speed) = 0.34; *M* (accuracy) = 0.03, SD (accuracy) = 0.24]. Mean reaction times were 683 ms (SD = 0.08) in the speed condition and 1,312 ms (SD = 0.40) in the accuracy condition. Thus, in the speed condition, participants were significantly faster, *t*(58) = −8.45, *p* < 0.01, *d* = 2.18.

#### Parameter estimation and model fit

We used the same parameter estimation procedure as in Experiment 1. We excluded 57 trials (<1 %). With fast-dm, only one individual model had to be excluded, because the KS test indicated a significant difference between the empirical and the predicted distribution. A binomial test revealed that the probability of finding one or more significant tests by chance was *p* = 0.19. Thus, the results indicate that overall, the model fitted the data well.

With DMAT, the Chi-square test showed good model fit for 53 models and poor model fit for the remaining seven individual models. We only included models with sufficient fit (i.e., *p* > 0.05). Since some participants made very few mistakes, we encountered several warnings with DMAT.

#### Parameter analyses with fast-dm

Like in Experiment 1, drift rates for new items were significantly steeper than those for old items (speed: *M* (old) = 0.04, SD (old) = 0.83, *M* (new) = 0.17, SD (new) = 0.11, *t*(29) = −6.96, *p* < 0.01, *d* = 1.27; accuracy: *M* (old) = 0.05, SD (old) = 0.56, *M* (new) = 0.09, SD (new) = 0.76, *t*(29) = 3.10, *p* < 0.01, *d* = 0.57). Again, we conducted an independent-samples *t* test for each parameter. In both conditions, the bias parameter *z*/*a* was significantly biased towards the old response (speed: *t*(29) = 3.20, *p* < 0.01, *d* = 0.60; accuracy: *t*(29) = 4.25, *p* < 0.01, *d* = 0.78).

**Fig. 4:**
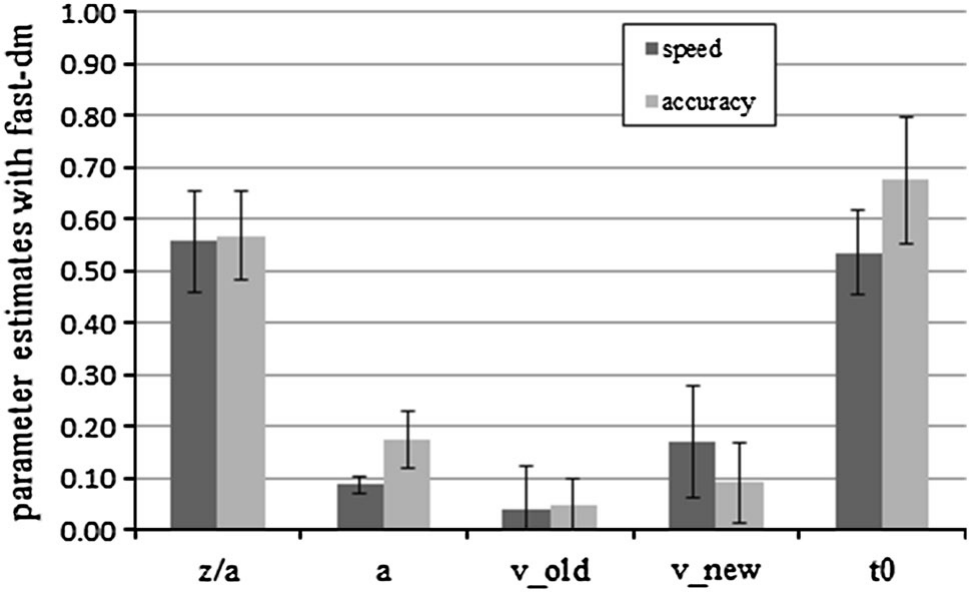
Mean fast-dm parameter estimates for speed and accuracy conditions in Experiment 2. *Bars* represent standard deviation. We show the absolute values of the drift rates. *z*/*a* represents the bias parameter, *a* the threshold parameter, *v*_old_ the drift rate for old items, *v*_new_ the drift rate for new items, and *t*_0_ the response-time constant

Consistent with the hypothesis, the threshold parameter *a* was significantly higher in the accuracy condition than in the speed condition, *M* (speed) = 0.09, SD (speed) = 0.02, *M* (accuracy) = 0.17, SD (accuracy) = 0.06, *t*(33.44) = −8.37, *p* < 0.01, *d* = 2.13. We also found significant differences in the drift rate for new items, *M* (speed) = −0.17, SD (speed) = 0.11, *M* (accuracy) = −0.09, SD (accuracy) = 0.08, *t*(58) = −3.30, *p* < 0.01, *d* = 0.96, and in the response-time constant *t*_0_, *M* (speed) = 0.54, SD (speed) = 0.08, *M* (accuracy) = 0.67, SD (accuracy) = 0.12, *t*(58) = −5.10, *p* < 0.01, *d* = 1.19. The effect size (measured as Cohen’s *d*) was about twice as large for the threshold parameter *a* as for drift rate *v*_new_ and response-time constant *t*_0_. However, all effect sizes represent large effects according to Cohen (1988). No other difference was significant (all *p* > 0.05). Averaged mean parameter estimates are shown in Fig. 4.

#### Parameter analyses with DMAT

**Fig. 5:**
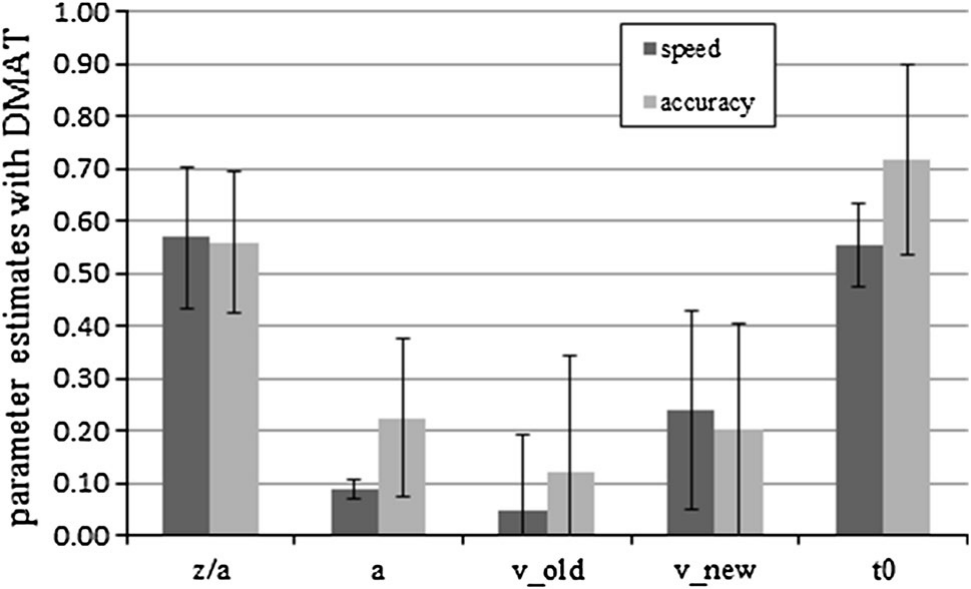
Mean DMAT parameter estimates for speed and accuracy conditions in Experiment 2. *Bars* represent standard deviation. We show the absolute values of the drift rates. *z*/*a* represents the bias parameter, *a* the threshold parameter, *v*_old_ the drift rate for old items, *v*_new_ the drift rate for new items, and *t*_0_ the response-time constant

Drift rates for new items were significantly steeper than those for old items only in the speed condition (speed: *M* (old) = 0.04, SD (old) = 0.15, *M* (new) = 0.26, SD (new) = 0.21, *t*(22) = −3.85, *p* < 0.01, *d* = 0.80; accuracy: *M* (old) = 0.12, SD (old) = 0.22, *M* (new) = 0.20, SD (new) = 0.20, *t*(29) = −1.33, *p* = 0.19, *d* = 0.35). Again, consistent with the hypothesis, the threshold parameter *a* was significantly higher in the accuracy condition than in the speed condition, *M* (speed) = 0.09, SD (speed) = 0.02, *M* (accuracy) = 0.21, SD (accuracy) = 0.15, *t*(30.721) = −4.79, *p* < 0.01, *d* = 1.24. We also found significant differences in the response-time constant *t*_0_, *M* (speed) = 0.55, SD (speed) = 0.09, *M* (accuracy) = 0.72, SD (accuracy) = 0.18, *t*(43.91) = −4.33, *p* < 0.01, *d* = 1.15. No other difference was significant (all *p* > 0.05). Averaged mean parameter estimates are shown in Fig. 5.

#### Parameter analyses with EZ

**Fig. 6:**
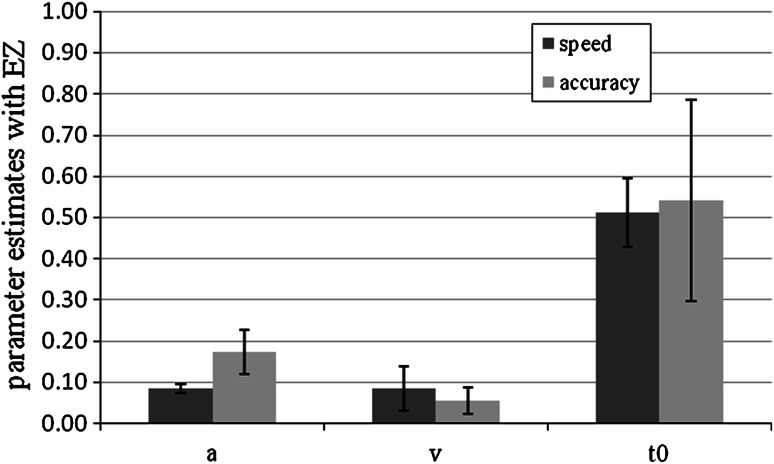
Mean EZ parameter estimates for speed and accuracy conditions in Experiment 2. *Bars* represent standard deviation. *a* represents the threshold parameter, *v* the drift rate for correct answers, and *t*_0_ the response-time constant

For the analysis with the EZ-diffusion model, we computed only one drift rate for old and new items by coding the old and new responses as correct and incorrect responses. The analysis showed significant differences in drift rate, *M* (speed) = 0.08, SD (speed) = 0.05, *M* (accuracy) = 0.05, SD (accuracy) = 0.03, *t*(46.94) = 2.58, *p* = 0.01, *d* = 0.73, and in the threshold parameter, *M* (speed) = 0.08, SD (speed) = 0.01, *M* (accuracy) = 0.17, SD (accuracy) = 0.05, *t*(31.42) = −8.66, *p* < 0.01, *d* = 2.50. This concurs with the fast-dm results except that there was no difference in the response-time constant *t*_0_. Averaged mean parameter estimates are shown in Fig. 6.

### Discussion

We conducted Experiment 2 to validate the interpretation of the threshold parameter *a*. The threshold parameter is a measure of conservatism. It defines how much information a participant needs to give an answer. It also describes the speed–accuracy trade-off. If the thresholds lie close together, the answer is given fast, but it is less likely to be accurate. Thresholds that lie far apart more likely lead to accurate answers, but at the cost of speed. We told participants in different experimental conditions either to respond very accurately or to respond very fast.

As predicted, we found that the threshold parameter was significantly higher in the accuracy condition with all parameter-estimation methods. With fast-dm and DMAT, we also found a higher response-time constant *t*_0_ in the accuracy condition. Because of the time pressure in the speed condition, participants carried out the non-decisional components of the task (such as motor response) faster than participants in the accuracy condition. This result was also obtained by Voss et al. (2004) and is easy to explain in psychological terms without questioning the discriminant validity of the parameters. However, with the EZ-diffusion model analysis this parameter did not show significant differences.

The significant difference in drift rates for new items, found with fast-dm and EZ, cannot easily be explained. Differences in drift rates for speed–accuracy manipulations were also found by Vandekerckhove, Tuerlinckx, and Lee (2008) and Starns et al. (2012). Both studies did not use the original version of the diffusion model but extended versions. Vandekerckhove et al. allowed for non-linear drift rates. Starns et al. allowed for different standard deviations s_*ν*_ of the drift rates for old and new items in a recognition-memory test. Heathcote and Love (2012) found that a speed–accuracy manipulation affected rate variability in the linear ballistic accumulator model which can be seen as a simplified diffusion model.

However, there is no apparent reason why a speed–accuracy manipulation should affect only the drift rate for new items, but not the drift rate for old items. One speculation is that under accuracy instructions, the original model proposed by Ratcliff (1978) may be appropriate which assumes an exhaustive search of the memory set for new items and waits until the last diffusion process stops, whereas under speed conditions, participants might be satisfied with the outcome of a small sample of diffusion processes. For old items, the fastest diffusion process is sufficient for a choice. The estimated average drift rate would thus be affected for new items, but not for old ones.

Unexpectedly, the speed–accuracy manipulation did not affect SDT’s sensitivity parameter *d*′. However, the experiment did show the expected effect of the speed–accuracy manipulation on parameter *a* which measures speed–accuracy calibration. The experiment is thus still valuable for showing the validity of the diffusion model.

If one compares effect sizes, all effects can be classified as “large” effects according to Cohen (1988). However, the effect on the threshold parameter *a* was much larger than the other effects, thus supporting strong convergent validity and only a mild threat of discriminant validity. Again, DMAT showed sufficient convergent and discriminant validity.

## Experiment 3

The purpose of Experiment 3 was to test the validity of the drift-rate parameter *v*. To affect drift rate, we varied encoding by manipulating the frequency of item presentation. We presented half of the items once and half of the items twice. According to Ratcliff (1978), two presentations will lead to two memory traces that both compete in a diffusion race, and the faster process “wins”, leading to higher accuracy and higher drift rates. In contrast to Experiments 1 and 2, we used a within-subjects manipulation. Ratcliff et al. (2004) used a similar manipulation, but they fixed the other model parameters between the conditions and compared the results for young and older adults. They showed that the model was able to capture changes in response-time distributions and accuracy as a function of word frequency and number of repetitions with only drift rate changing. To achieve stable parameter estimates in the three conditions of our within-subjects design, longer learning lists were necessary. We used pictures in this study, which are easier to remember than words (Paivio, 1971), to avoid floor effects due to longer lists. Also, the use of pictures in this experiment allowed us to generalize findings to other materials. Participants received no feedback. Items that were presented twice should have a higher drift rate than items that were presented only once. This manipulation should not influence the other parameters.

### Methods

#### Participants

Twenty-eight students (24 female; *M* age of 24.11 years with a range of 20–35 years) of the University of Düsseldorf participated for course credit or monetary payment. There were between one and four participants in each session.

#### Materials

The stimuli were 275 line drawings of simple objects or animals from Snodgrass and Vanderwart (1980) and the free online resource of Szekely et al. (2004). They were selected such that there were no two items of one subcategory (e.g., insects).

#### Design and procedure

Number of presentations was manipulated within participants (not presented, presented once, presented twice). For each participant, 270 items were randomly and equally assigned to the three conditions. The items in the conditions “presented once” and “presented twice” were each randomly split into 15 blocks of 6 items. The blockwise randomization ensured that there were at least 6 items and at most 16 items between the first and the second presentation of twice-presented items. Each item was presented for 1,500 ms in the middle of the screen. The items were preceded by six pictures that served as primacy buffer and were the same for all participants.

A 20-min retention interval followed, during which participants played Solitaire. At test, items were drawn in random order from all lists. They were asked to decide as quickly and as accurately as possible if items had been presented during study or not. They received no feedback. Assignment of marked keys to responses was the same as in Experiments 1 and 2.

### Results

#### Performance measures

Mean hit rates differed significantly between items presented once and items presented twice, *M* (presented once) = 0.58, SD (presented once) = 0.18, *M* (presented twice) = 0.77, SD (presented twice) = 0.18, *t*(27) = −9.34, *p* < 0.01, *d* = 1.81. The false-alarm rate was 0.13 (SD = 0.08) for not presented items. Mean reaction times were 818 ms (SD = 0.11) for items that were not presented, 829 ms (SD = 0.11) for items presented once, and 781 ms (SD = 0.09) for items presented twice, a significant effect, *F*(2,54) = 8,86, *p* < 0.01, *η*_*p*_^2^ = 0.25.

#### Parameter estimation and model fit

Again, parameters were estimated with fast-dm, DMAT, and EZ. For each participant, we calculated three separate models, one for each condition. Thus, each model was based on 90 trials. We excluded a total of 24 trials (<1 %) according to the same criteria as in the other experiments. We estimated seven parameters: the mean bias parameter *z*, the mean upper threshold *a*, the mean drift rate *v*, mean response-time constant *t*_*0*_ and the ranges of bias parameter *s*_*z*_ and response-time constant *s*_*t*_ as well as the standard deviation of the drift rates *s*_*v*_. The KS test used in fast-dm showed a good fit (p > 0.05) for all models. The Chi-square test used in DMAT showed a good fit for all calculated models. However, for some participants, DMAT failed to estimate parameter values in some conditions due to too few error responses. Thus, we included only participants with given parameter estimates in all conditions, resulting in only 11 participants.^1^

#### Parameter analyses with fast-dm

To test the influence of the manipulation we conducted repeated-measures ANOVAs for each parameter, with number of presentations as the independent variable. For the drift rates, we used the absolute values. As predicted, there were significant differences in the drift rates, *M* (not presented) = −0.21, SD (not presented) = 0.09, *M* (presented once) = 0.05, SD (presented once) = 0.08, *M* (presented twice) = 0.14, SD (presented twice) = 0.12, *F*(2,54) = 22.68, *p* < 0.01, *η*_*p*_^2^ = 0.46. As Helmert contrasts revealed, the difference between old versus new items was significant, *F*(1,27) = 19.99, *p* < 0.01. The difference between once- and twice-presented items was significant as well, *F*(1,27) = 30.52, *p* < 0.01.

Unexpectedly, the bias parameter also showed significant differences, *M* (not presented) = 0.41, SD (not presented) = 0.10, *M* (presented once) = 0.49, SD (presented once) = 0.14, *M* (presented twice) = 0.56, SD (presented twice) = 0.12, *F*(1.49,40.23) = 15.10, *p* < 0.01, *η*_*p*_^2^ = 0.36, *df*s Greenhouse–Geisser corrected. The more often the items were presented (not, once, twice) the higher was the bias-parameter *z*/*a*. The response-time constant *t*_0_ also showed significant differences, *M* (not presented) = 0.63, SD (not presented) = 0.07, *M* (presented once) = 0.60, SD (presented once) = 0.06, *M* (presented twice) = 0.59, SD (presented twice) = 0.07, *F*(2,54) = 6.18, *p* < 0.01, *η*_*p*_^2^ = 0.19. As expected, the threshold parameter *a* did not differ significantly between the conditions.

**Fig. 7:**
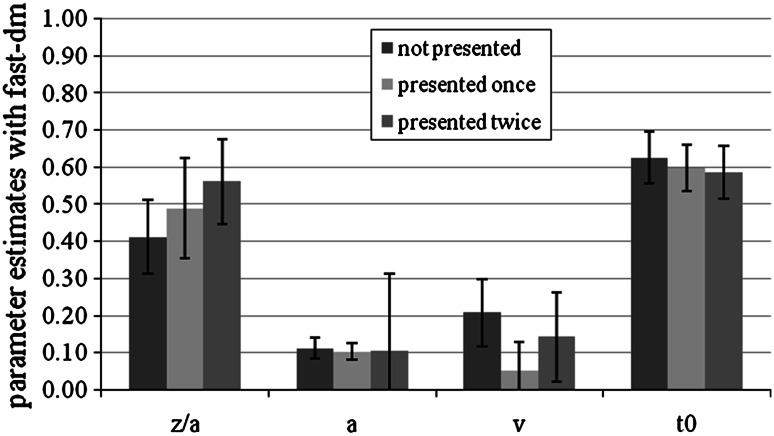
Mean fast-dm parameter estimates for not presented items, items presented once and items presented twice conditions in Experiment 3. *Bars* represent standard deviation. We show the absolute values of the drift rates. *z*/*a* represents the bias parameter, *a* the threshold parameter, *v* the drift rate, and *t*_*0*_ the response-time constant

For Experiment 3, we also performed analyses in which we fixed all parameters between conditions, except one which was allowed to vary (either *z*, *a*, *v*, or *t*_0_). This was recommended by Ratcliff (1978) and is possible for within-subject designs only. We expected that the model in which *v* was free to vary would show the best model fit. The models that allowed the threshold parameter *a* or the response-time constant *t*_0_ to vary, did not fit the data. We found satisfactory model fit only for models that allowed either the drift rate *v* or the starting point *z* to vary. The model that allowed for drift rate variation had a model fit that was more than four times better than the model that allowed for variation of the starting point *z*. Averaged mean parameter estimates are shown in Fig. 7.

#### Parameter analyses with DMAT

As predicted, there were significant differences in the drift rates, *M* (not presented) = −0.48, SD (not presented) = 0.41, *M* (presented once) = 0.17, SD (presented once) = 0.38, *M* (presented twice) = 0.69, SD (presented twice) = 0.69, *F*(1.74,17.44) = 4.49, *p* = 0.03, *η*_*p*_^2^ = 0.31, *df*s Greenhouse–Geisser corrected. As Helmert contrasts revealed, the difference between old versus new items was not significant, *F*(1,10) = 0.07, *p* = 0.79. However, as predicted, the difference between once- and twice-presented items was significant, *F*(1,10) = 12.30, *p* < 0.01.

Unexpectedly, the bias parameter also showed significant differences, *M* (not presented) = 0.27, SD (not presented) = 0.27, *M* (presented once) = 0.57, SD (presented once) = 0.20, *M* (presented twice) = 0.61, SD (presented twice) = 0.18, *F*(2,20) = 6.99, *p* = 0.01, *η*_*p*_^2^ = 0.41 due to the difference between non-presented items and presented items, *F*(1,10) = 8.53, *p* = 0.02. There was no difference between items presented once and items presented twice, *F*(1,10) = 0.59, *p* = 0.47.

The threshold parameter *a* also showed significant differences, *M* (not presented) = 0.65, SD (not presented) = 0.72, *M* (presented once) = 0.12, SD (presented once) = 0.04, *M* (presented twice) = 0.12, SD (presented twice) = 0.03, *F*(1.01,10.05) = 6.02, *p* = 0.03, *η*_*p*_^2^ = 0.38, *df*s Greenhouse–Geisser corrected. Again, this difference resulted due to the difference between non-presented items and presented items, *F*(1,10) = 6.04, *p* = 0.03. There was no difference between items presented once and items presented twice, *F*(1,10) = 0.59, *p* = 0.81. As expected, the response-time constant *t*_0_ did not differ significantly between the conditions.

**Fig. 8:**
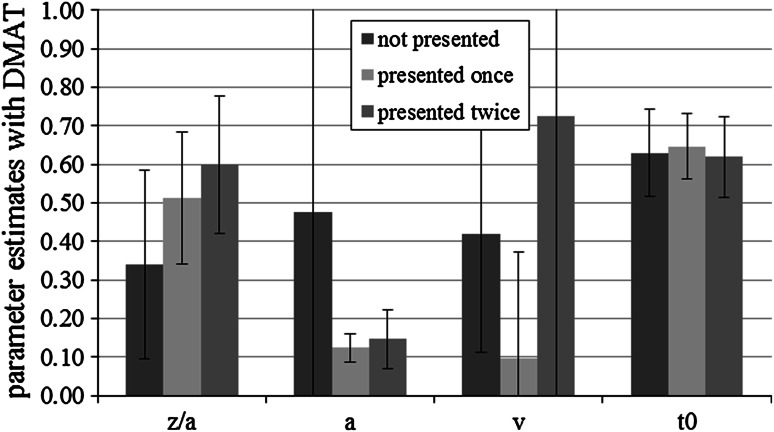
Mean DMAT parameter estimates for not presented items, items presented once and items presented twice conditions in Experiment 3. *Bars* represent standard deviation. We show the absolute values of the drift rates. *z*/*a* represents the bias parameter, *a* the threshold parameter, *v* the drift rate, and *t*_0_ the response-time constant

With DMAT, we also performed analyses in which we fixed all parameters between conditions, except one which was allowed to vary (either *z*, *a*, *v*, or *t*_0_). The model that allowed for drift-rate variation had––with four exceptions––the best (and acceptable) model fit. Averaged mean parameter estimates are shown in Fig. 8.

#### Parameter analyses with EZ

**Fig. 9:**
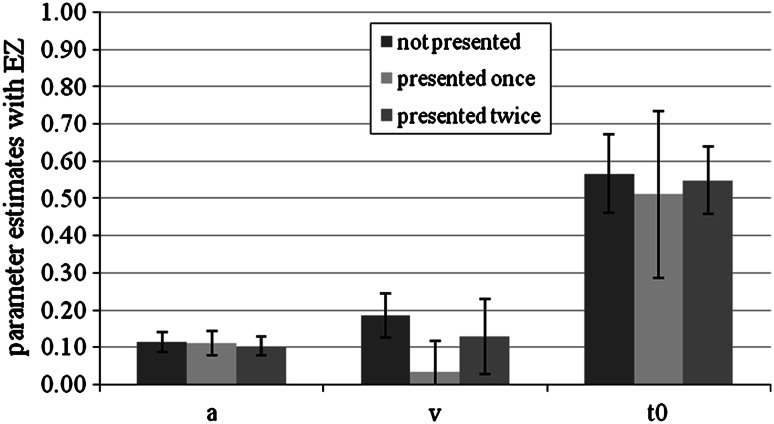
Mean EZ parameter estimates for not presented items, items presented once and items presented twice conditions in Experiment 3. *Bars* represent standard deviation. We show the absolute values of the drift rates. *a* represents the threshold parameter, *v* the drift rate, and *t*_0_ the response-time constant

For two participants, the EZ-diffusion model could not be calculated in all conditions due to perfect accuracy. For the remaining 26 participants, the EZ-diffusion model showed significant differences in (absolute) drift rate only, *M* (not presented) = 0.19, SD (not presented) = 0.06, *M* (presented once) = 0.03, SD (presented once) = 0.08, *M* (presented twice) = 0.13, SD (presented twice) = 0.10, *F*(1.41,35.17) = 33.19, *p* < 0.01, *η*_*p*_^2^ = 0.57 (*df*s Greenhouse–Geisser corrected). Helmert contrasts revealed that new and old items were significantly different, *F*(1,25) = 24.54, *p* < 0.01. Additionally, items presented once and items presented twice differed significantly, *F*(1,25) = 73.54, *p* < 0.01. Threshold parameter *a* and response-time constant *t*_0_ did not show significant differences. Averaged mean parameter estimates are shown in Fig. 9.

### Discussion

The third experiment was designed to validate the interpretation of the drift-rate parameters. For the recognition-memory paradigm, drift rates derived from the diffusion model are supposed to be pure measures of memory. In the previous experiment, we already found different drift rates for old and new items. In this experiment, we had three types of test items: items that had been presented once during study, items that had been presented twice during study, and new items. We calculated independent models for the three types of stimuli. The number of presentations should affect the drift rate for old items. We had no hypothesis regarding the drift rate for new items.

Compatible with the prediction, we found significant differences in drift rates, which were not merely due to differences between old and new items. The drift rates for items presented once and items presented twice showed significant differences as well. Drift rates for items presented twice were more than twice as high as drift rates for items presented only once.

With fast-dm, there were also significant differences in the response-time constant *t*_0_ and the bias parameter *z*/*a*. These results were contrary to predictions, but are in line with a finding by Criss (2010) who found a correlation between bias parameter *z* and drift-rate parameter *v*. The difference in the response-time constant *t*_0_ can be explained easily. Differences in *t*_0_ between the conditions are probably due to enhanced encoding. Items that have been presented before are more readily accessible for encoding.

For the bias parameter *z*/*a* and the response-time constant *t*_0_, the absolute differences were quite small (see Fig. 9). The effect of the frequency manipulation on *v* was considerably larger than those on the other two parameters.

With DMAT, there were also significant differences in the bias parameter and the threshold parameter. The difference in the bias parameter cannot be explained within the diffusion model. Since all pictures were randomly distributed across the three learning conditions, there cannot be systematic differences between stimuli to account for different starting points of the diffusion process. Hence, differences in estimated bias must be due to a misspecification of the model or inaccuracies in the estimation procedure. Since both a higher drift rate for old items and a bias in favor of old items predict faster RTs for correct “old” responses, the parameter estimation procedures may attribute some of the observed differences to both processes. Hence, this effect on bias does not necessarily invalidate the diffusion model, but it may hint to limitations of the estimation procedures to fully disentangle the effects of different cognitive processes from the given data structure.

Differences in the threshold parameters could perhaps be accounted for if one assumed a dynamic interaction of drift rate and threshold: suppose a participant has a certain speed–accuracy optimum. In trials with very quick drift, she could “afford” to spend some extra time to increase accuracy even further, leading to a wider estimated spacing of thresholds. It is unclear, however, if such a dynamic extension of the model is theoretically desirable and/or practically manageable. However, in contrast to the bias effect, the threshold effect can at least potentially be explained within the diffusion model framework. Unfortunately, we had to exclude more than half of the participants from the DMAT analyses. This led to a very small sample size and thus to very low statistical power. Still, we found the predicted parameter differences to be significant.

The EZ-diffusion model analysis did not show significant differences in the *t*_*0*_, most likely due to the small absolute difference in this parameter between conditions. A difference in the bias parameter *z*/*a* cannot be detected by the EZ-diffusion model, of course. The restricted model versions showed best model fit for the model that allowed the predicted parameter *v* to vary between the conditions.

## General discussion

In three experiments, we explored the convergent and discriminant validity of the central diffusion-model parameters *v*, *z*, and *a* by manipulating variables that were each selected to affect a corresponding single process represented in the model. With respect to convergent validity, all tests were clearly positive. That is, in each instance, the experimental manipulation had a large effect on the target parameter in the expected direction.

With respect to discriminant validity, the results were less clear-cut. All manipulations had some side effects on other model parameters as well. In two cases, this was psychologically meaningful, namely the effect of the speed instruction on the response-time constant *t*_0_ in Experiment 2 and the effect of the number of presentations on *t*_0_ in Experiment 3. However, other effects were harder to reconcile psychologically, namely the effect of the number of presentations (Experiment 3) on the bias parameter *z*/*a* and on the threshold parameter *a*. Whereas the threshold effect may be explained within the diffusion model by invoking additional assumptions (see above), the bias effect is clearly unexplainable within the model.

For the effect of speed versus accuracy instructions on the drift rate only for new items (Experiment 2), a possible explanation entails Ratcliff’s (1978) original assumption of an exhaustive search for negative responses in recognition. It is possible that participants dispense with exhaustiveness under speed instructions and base their decision on a subset of parallel processes. This, in turn, leads to faster drift rates.

Comparing the different methods for parameter estimation, our conclusion is similar to those of van Ravenzwaaij and Oberauer (2009). Fast-dm showed smaller differences between conditions––especially for drift rates in Experiments 1 and 3. DMAT gave error messages when the number of trials was extremely small. EZ was very robust but cannot estimate all parameters.

Hence, if one assumes that the experimental manipulations used in our experiments selectively influenced the respective cognitive processes, one must conclude that the measures derived via parameter estimation––at least as estimated with the fast-dm method––are not entirely “process pure.” Analysis with the EZ-diffusion model showed similar results, except that it did not find differences in *t*_0_ that were psychologically plausible. DMAT showed discriminant validity for Experiment 1 and Experiment 2, but failed to do so for Experiment 3. Parameters *t*_0_, *z*, and *v* all represent cognitive processes that affect decision times. However, when they must be recovered from a noisy response-time distribution, their respective influences presumably cannot be clearly separated. This does not necessarily undermine the validity of the diffusion model in certain applications, though, given specific conditions discussed below. To put our findings into perspective, the reader is reminded that in every case, the effect sizes were considerably larger for the target parameters than for the side effects. Hence, the lion’s share of variation in the data could always be attributed to the correct parameter, and it may, thus, be warranted to conclude that the model has some discriminant validity, although it is rather weak. Voss et al. (2004) concluded that their findings supported the validity of the diffusion model. However, their results were more straightforward in that their manipulations only affected the hypothesized parameters (except for the response handicap condition which we did not test).

We recommend fixing parameters across conditions whenever this can be justified for strong theoretical or procedural reasons and to estimate freely only the parameters of interest in a study. If the parameter restrictions are not justified, this should reveal itself in a misfit of the model. To test this with our data, we calculated different restricted model versions in Experiment 3. These analyses showed that only the psychologically plausible restrictions provided good model fit, and models with implausible restrictions failed to fit the data.

The diffusion model is a very useful tool to disentangle processes in binary choice tasks such as recognition. It goes beyond SDT and threshold models in providing a dynamic process description. Furthermore, it allows us to disentangle memory performance further into objective (drift rate) and subjective (threshold and starting point) components of performance and to model speed–accuracy trade-offs (Wagenmakers, 2009). By incorporating response times, it uses a richer database than SDT or threshold models. The results of our experiments show that the model parameters can largely be used as valid measures of the proposed underlying processes with some caution regarding strong claims about discriminant validity. Whether this problem requires an extended theory, more robust estimation procedures, or sophisticated methods of cleaning response-time data, remains an issues for further research.

## Footnotes

1 Excluded participants did not differ from included participants in terms of age, hit and false-alarm rates, and mean reaction time.
